# A multiband perfect absorber based on hyperbolic metamaterials

**DOI:** 10.1038/srep26272

**Published:** 2016-05-18

**Authors:** Kandammathe Valiyaveedu Sreekanth, Mohamed ElKabbash, Yunus Alapan, Alireza R. Rashed, Umut A. Gurkan, Giuseppe Strangi

**Affiliations:** 1 Department of Physics, Case Western Reserve University, 10600 Euclid Avenue, Cleveland, OH, 44106 (USA); 2 Case Biomanufacturing and Microfabrication Laboratory, Mechanical and Aerospace Engineering Department, Case Western Reserve University, Cleveland, OH, 44106 USA; 3 Biomedical Engineering Department, Case Western Reserve University, Cleveland, OH, 44106 USA; 4 Department of Orthopaedics, Case Western Reserve University, Cleveland, OH, 44106 USA; 5 Advanced Platform Technology Center, Louis Stokes Cleveland Veterans Affairs Medical Center, Cleveland, OH, 44106 USA; 6 Department of Physics and CNR-NANOTEC UOS of Cosenza, Licryl Laboratory, University of Calabria, 87036 - Rende (Italy)

## Abstract

In recent years, considerable research efforts have been focused on near-perfect and perfect light absorption using metamaterials spanning frequency ranges from microwaves to visible frequencies. This relatively young field is currently facing many challenges that hampers its possible practical applications. In this paper, we present grating coupled-hyperbolic metamaterials (GC-HMM) as multiband perfect absorber that can offer extremely high flexibility in engineering the properties of electromagnetic absorption. The fabricated GC-HMMs exhibit several highly desirable features for technological applications such as polarization independence, wide angle range, broad- and narrow- band modes, multiband perfect and near perfect absorption in the visible to near-IR and mid-IR spectral range. In addition, we report a direct application of the presented system as an absorption based plasmonic sensor with a record figure of merit for this class of sensors.

Recently, subwavelength nanostructures have received considerable attention because their absorption properties can be controlled^1^. There are two main classes of perfect absorbers determined by their spectroscopic features: (i) broadband absorbers with a wide frequency spectrum^2^,^3^,^4^,^5^ and (ii) spectrum-selective absorbers with a narrowband spectrum^6^,^7^,^8^,^9^,^10^,^11^. In recent years, metamaterials have been investigated in terms of absorption enhancement because of their ability to achieve almost perfect absorption with high efficiency^2^,^3^,^4^,^5^,^6^,^7^,^8^. However, these complex structures achieve narrowband unit absorption at infrared and THz frequencies, but not at visible and near infrared (NIR) frequencies. In addition, grating-based metamaterials can efficiently enhance the absorption and are relatively easy to fabricate, but perfect absorption is only possible at a resonant wavelength by using impedance matching to minimize the reflectance^9^,^10^,^11^. Most of the works on metamaterial perfect absorbers (MPAs) focused on broadening the absorption band by using different strategies such as overlapping multi-resonant impedance matching absorbers^12^,^13^ or by using a plasmonic grating absorber with metallic array of trapezoidal shaped gratings^14^. Recently, a metamaterial absorber based on gold nanorod-liquid crystal hybrid has been proposed for tunable total absorption at infrared frequencies^15^. On the other hand, very recently other efforts have been dedicated to create ultra-narrow band absorbers that may have many applications as absorption filters, narrow band thermal emitters, and plasmonic biosensors^16^,^17^. In addition, significant research has focused on creating polarization independent absorption for both TM and TE polarizations using complicated designs to overcome this issue, which is inherent to impedance matching MPA mechanism^4^,^18^. Furthermore, visible light absorption has been recently demonstrated^9^,^10^,^11^,^14^ nonetheless have been achieved perfect absorption due to the fabrication difficulties related to low throughput in high frequency regimes especially for purposes that require large absorption areas such as energy harvesting applications and thermo-photovoltaics.

Hyperbolic metamaterials (HMMs) are promising nanostructured metamaterials with blackbody behavior for both broadband and narrowband spectra. HMMs are exquisitely simple nanostructures that can be fabricated using conventional thin film deposition techniques by depositing thin metal-dielectric multilayers in the effective medium regime^19^,^20^,^21^. This results in indefinite k-space dispersion of hyperbolic type, therefore supporting high-k modes and divergent photonic density of states. In order to realize hyperbolic metamaterials at visible frequencies, gold and silver along with dielectric such as Al_2_O_3_ or TiO_2_ forms an ideal choice. It is also possible to tune the hyperbolic dispersion in the visible region by selecting appropriate combination of metal (gold and silver) and dielectric (Al_2_O_3_ and TiO_2_) as well as with appropriate filling fraction^19^. HMMs have been theoretically predicted as electromagnetic absorbers for scattered fields. This was experimentally demonstrated by placing scatterers on top of HMM, showing enhanced absorption but it was neither perfect nor narrow-band absorption^22^,^23^,^24^. A graphene-based hyperbolic metamaterial has also been proposed for perfect absorption at terahertz frequencies^25^. Although HMMs are designed by interlocking low loss dielectric materials and highly reflective noble metals, the strong absorption of light is realized via the indefinite dispersion of such meta-structures. This unusual hyperbolic dispersion results in a divergence of the photonic density of states, and it leads to a dramatic increase in the absorption of incident photons that get absorbed into the propagating modes of the hyperbolic medium. Effectively, Type II HMMs showing overall positive permittivity in the bulk are able to confine the electromagnetic field in a very small volume while allowing it to propagate at the same time in the form of bulk plasmon polaritons (BPPs)^26^,^27^,^28^. We have previously investigated the existence of such BPPs in our grating coupled HMM (GC-HMM) system^29^,^30^ and the spontaneous emission rate enhancement of fluorescent molecules embedded GC-HMM, which experimentally prove the existence of high photonic density of states^31^.

Here, we show that the GC-HMM opens new venues for engineering electromagnetic absorption based on the excitation of BPPs. We explored an alternative route for perfect absorption using GC-HMMs that offer polarization independent behavior, both broad-band and narrow-band absorption, wide angle range absorption and multiband perfect absorption for a wide spectral range from visible to infrared. In addition, we report on how our ultra-narrow band absorber can be used to realize a high-sensitivity absorption-based plasmonic sensor with an unprecedented high figure of merit.

## Results

### Design and fabrication of the GC-HMM absorbers

**Figure 1:**
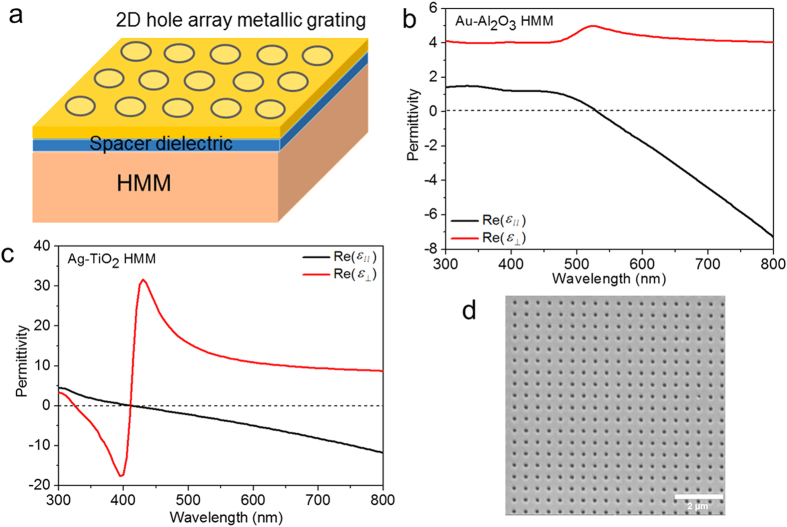
Design and characterization. (**a**) Schematic representation of grating coupled HMM. EMT-derived real parts of effective uniaxial permittivity components of (**b**) Au-Al_2_O_3_ HMM, and (**c**) Ag-TiO_2_ HMM. Au-Al_2_O_3_ HMM shows Type II hyperbolic dispersion at λ > 520 nm and Ag-TiO_2_ HMM shows both Type I (325 nm < λ > 410 nm) and Type II (λ > 410 nm) hyperbolic dispersion at different spectral regions. (**d**) SEM image of 2D metallic hole array diffraction grating on top of the HMM (period = 500 nm and hole diameter = 160 nm).

In order to realize both broad- and narrow-band absorbers, we designed and fabricated two different GC-HMMs (Fig. 1a); a palladium grating coupled silver-titanium dioxide HMM and a gold grating coupled gold-aluminum dioxide HMM (also see Supplementary Fig. S1). Since HMMs can be defined in the effective medium approximation framework, we have used effective medium theory (EMT) to design our planar metal-dielectric metastructures. The hyperbolic metastructures comprise a) 12 alternating layers of silver (Ag) and titanium dioxide (TiO_2_) thin films and b) 16 alternating layers of gold (Au) and aluminum dioxide (Al_2_O_3_) thin films. They are fabricated by the sequential deposition of TiO_2_ and Ag layers, and Al_2_O_3_ and Au layers on a glass substrate using electron beam evaporation of TiO_2_ and Al_2_O_3_ pallets and the thermal evaporation of Ag and Au pellets (see Methods). Variable-angle spectroscopic ellipsometry was used to determine the thicknesses of all layers (20 nm for Ag and TiO_2,_ 15 nm for Au, and 30 nm for Al_2_O_3_). The EMT-derived dielectric permittivity tensor components of the HMM structures are shown in Fig. 1b,c. In order to obtain the uniaxial permittivity values (*ε*_*ll*_ = *ε*_*x*_ = *ε*_*y*_ and *ε*_⊥_ = *ε*_*z*_), the experimentally determined permittivity values of Au, Ag, Al_2_O_3_ and TiO_2_ are used (see Supplementary Fig. S2). Spectroscopic ellipsometry measurements confirmed the behavior of the dielectric tensor components. According to Fig. 1b, the fabricated Au-Al_2_O_3_ HMM is a Type II HMM with dielectric permittivity tensor components *ε*_*ll*_ < 0and *ε*_⊥_ > 0, confirming the designed hyperbolic dispersion at optical frequencies >520 nm wavelength. However, Ag-TiO_2_ HMM shows both Type 1 and Type II behavior at different spectral regions. As evidenced from Fig. 1c,a Type 1 HMM window is present between 325 nm and 410 nm (*ε*_*ll*_ > 0 and *ε*_⊥_ < 0) and Type II behavior above 410 nm. At the transition wavelength of 410 nm, a strong discontinuity in *ε*_⊥_, passing from a high negative value to a high positive value, as well as *ε*_*ll*_ = 0. In addition, the imaginary part of *ε*_⊥_ showing a very sharp Lorentzian shape, peaked exactly at the transition wavelength (see Supplementary Fig. S3). The experimentally obtained reflectance spectra also demonstrate the existence of dielectric singularity in Ag-TiO_2_ HMM (see Supplementary Fig. S4). In short, by choosing proper metal and dielectric thicknesses (same thickness for both TiO_2_ and Ag), it is possible to create both behavior (Type I and Type II) in metal-dielectric metastructures, also known as epsilon near zero and pole (*ε*_*NZP*_) HMM^32^,^33^,^34^.

**Figure 2:**
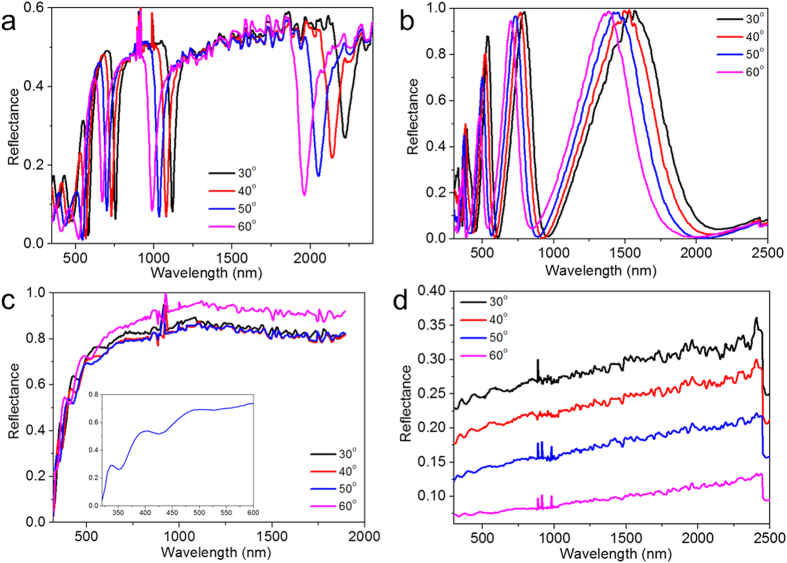
Reflectance spectra at different angles of incidence for (**a**) Au GC-Au/Al_2_O_3_ HMM, (**b**) Pd GC-Ag/TiO_2_ HMM, (**c**) 20 nm thick Au grating on an Al_2_O_3_/ glass and (**d**) 8 nm thick Pd grating on a TiO_2_/ glass.

To excite both surface and bulk plasmon modes of both HMMs, we designed and fabricated GC-HMMs combining a metallic diffraction grating (2D) and HMM. We used electron-beam lithography to pattern the diffraction grating on top of the HMMs (see Methods). To make both broad and narrow band absorbers, we directly deposited thin films of Pd (thickness = 8 nm) on the patterned surface of Ag-TiO_2_ HMMs and Au (thickness = 20 nm) on the patterned surface of Au-Al_2_O_3_ HMMs. The gratings have an average period of 500 nm, whereas the average hole diameter is 160 nm and the height is ~120 nm (Fig. 1d). In order to avoid direct contact between the grating and the HMM, a thin spacer layer (10 nm TiO_2_ for Ag-TiO_2_ HMM and 10 nm Al_2_O_3_ for Au-Al_2_O_3_ HMM) was deposited between them. Spectroscopic ellipsometry was used to characterize the fabricated GC-HMMs. The reflectance spectra of both Au GC-HMM and Pd GC-HMM are shown in Fig. 2a,b, respectively. In Fig. 2a, the reflectance minima obtained in the elliptical (λ < 520 nm) and the hyperbolic (λ > 520 nm) regions representing the surface plasmon polaritons (SPPs) and highly-confined bulk plasmon polaritons (BPPs) of Au GC-HMM, respectively^29^. However, all the modes obtained in the case of Pd GC-HMM (Fig. 2b) are BPPs because the hyperbolic dispersion of the Ag-TiO_2_ HMM starts from 325 nm wavelength. To further confirm the existence of BPP modes in Au GC-HMM and Pd GC-HMM, two reference samples (20 nm thick Au grating on an Al_2_O_3_/glass and 8 nm thick Pd grating on a TiO_2_/ glass) were fabricated (see Supplementary Fig. S1) and the reflectance spectra of the reference samples were compared with GC-HMMs. The reflectance spectra of the reference sample of Au GC-HMM is given in Fig. 2c, which shows two reflectance dip at the lower wavelengths (λ < 520 nm) representing the SPP modes of Au diffraction grating. Therefore, it is clear that the modes obtained in the hyperbolic spectral regions of Au GC-HMM are BPPs. However, the reference sample of Pd GC-HMM does not supports any modes as evidenced from Fig. 2d. Hence all the modes of Pd GC Ag-TiO_2_ HMM are BPPs. Also, the obtained modes of GC-HMMs are guided modes because they are blue shifted when the incident angle is increased from 30 to 60 degree. In short, multiple modes are possible for a given grating period that satisfy the momentum matching condition to bulk plasmon polaritons, once this takes place energy is transferred to these high-k modes.

The Pd GC-HMM provides broadband modes whereas Au GC-HMM provides narrowband modes. It shows that the choice of the grating metal results in a drastic change in the absorption properties of our system. Our observations indicate that the contrast between the grating effective permittivity and the bulk effective parallel permittivity determines the width of the modes. The lower the contrast in permittivity, the narrower the modes. It has been already demonstrated that if the effective average permittivity of the grating approaches the effective average permittivity of the plasmonic waveguide, the grating still exist while the waveguide starts to vanish^35^. According to our calculations, broadband spectra of Pd GC-HMM are due to the large contrast between the effective permittivity of the Pd grating and the Ag-TiO_2_ effective permittivity whereas the narrowband spectra of Au GC-HMM are due to the lower contrast between the Au grating and the Au-Al_2_O_3_ effective permittivity (see Supplementary Fig. S5). Specifically, 8 nm Palladium has higher contrast with respect to the effective parallel permittivity of the Ag-TiO2 HMM compared to the 20 nm Au grating in the Au-Al2O3 HMM. Also note that the mode bandwidth of both GC-HMMs increases with increasing wavelength. This is also due to the increase in contrast between the grating and the HMM structure as the wavelength increases (see Supplementary information).

### Broad-band perfect absorption

**Figure 3:**
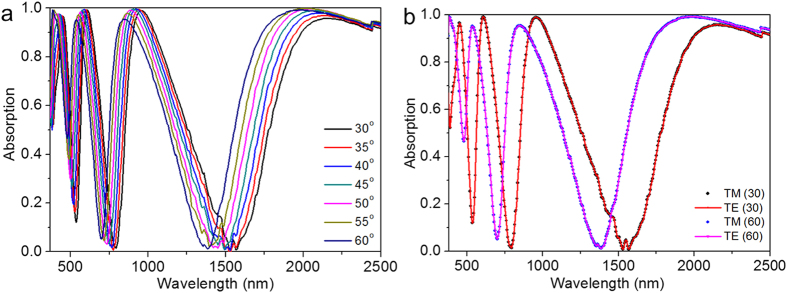
Polarization independent perfect absorption (**a**) Absorption spectra of Pd GC-Ag/TiO_2_ HMM at different angles of incidence, and (**b**) Absorption spectra of Pd GC-Ag/TiO_2_ HMM for TM and TE polarization. Wide angle, multiband and polarization independent broad perfect absorption is possible.

**Figure 4:**
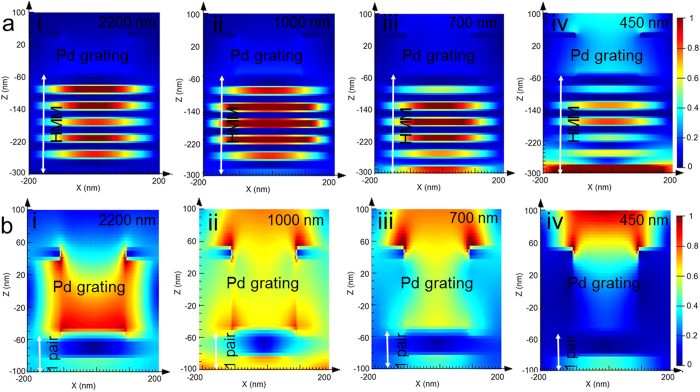
Cross-sectional electric field distribution maps of (**a**) Pd GC-Ag/TiO_2_ HMM at different BPP mode wavelengths, and (**b**) control sample (Pd GC-1 pair of Ag/TiO_2_) at the corresponding wavelengths.

First, we discuss the broad multiband absorption using Pd GC-HMMs. The absorption was obtained by assuming that the HMM surface is flat and any excited surface wave die out before re-scattering and thus its relation to transmission and reflection is given by A = 1-T-R. Angular transmission (see Supplementary Fig. S6) and reflection were measured using the variable angle spectroscopic ellipsometer. As shown in Fig. 3a, we thus obtain the absorption spectra of Pd-GC HMM for different angles of incidence, which exhibits above 98% absorption for all modes for a wide range of angle of incidence (30 to 60 degree). The perfect absorption (~100%) is obtained for different modes at different angles of incidence. The mode at 450 nm provides perfect absorption at 60° whereas modes at 600 nm, 950 nm and 2000 nm show at 35°, 40° and 55°, respectively. As mentioned earlier, the basic principle of the perfect absorption of GC-HMM is the indefinite hyperbolic k-space which dramatically increases the photonic density of states which in turns leads to enhancing the absorption of incident light inside the HMM in the form of BPP especially in the Type II region. Specifically, the density of states of HMMs are indefinite for all frequencies that satisfy the hyperbolic dispersion. By using sub-wavelength gratings it is possible to excite the BPPs depending on the predesigned grating period, which offers flexibility in engineering absorption. We performed simulations for the field distributions of BPP modes which clearly shows how the electromagnetic field propagates inside the HMM. In comparison with the field distributions of a control sample (8 nm thick Pd grating on 1 pair of Ag-TiO_2_) at TM polarization, Pd GC-HMM shows strong absorption of light inside HMM at the corresponding wavelength of BPP modes due to hyperbolic dispersion (Fig. 4). For all wavelengths, the field is concentrated inside the HMM for Pd GC-HMM (Fig. 4a), however, the field is mainly distributed around the grating in the case of control samples (Fig. 4b).

Another key feature of our design is that GC-HMM absorbers are polarization independent. In general, MPAs suffer from polarization dependence. The most common design for metamaterial is bi-anisotropic which results in polarization dependence. Overcoming this problem requires fabricating a combination of metamaterial absorbers rotated by 90 degrees. Our design of GC-HMM inherently exhibits a π/2 rotational symmetry due also to its 2D grating structure. However, if desired, using a 1D grating would break the rotational symmetry of the GC-HMM absorber and allow for polarization dependent absorption. Using a 2D grating, however, is necessary but not sufficient to realize complete polarization independence. For MPAs that rely on impedance matching only the absorption of the TM mode is left unaffected while the TE mode’s absorption decreases at higher angles of incidence. This is because the magnetic field creates parallel and antiparallel currents that generate the desired left handed magnetic response of impedance matching MPAs. For TE modes, the magnetic field lies in the same plane as the propagation k vector, and at higher angles the component along the plane decreases^36^. Our results, presented in Fig. 3b, using Pd GC-HMM clearly show that such effect does not occur since the TE and TM polarization has the exact same mode profile and absorption intensity for all angles. The field distributions of Pd GC-HMM for both (TM and TE) polarizations also confirm the polarization independent absorption behavior (see Supplementary Fig. S7).

### Ultra-narrowband near perfect absorption and plasmonic sensor

**Figure 5:**
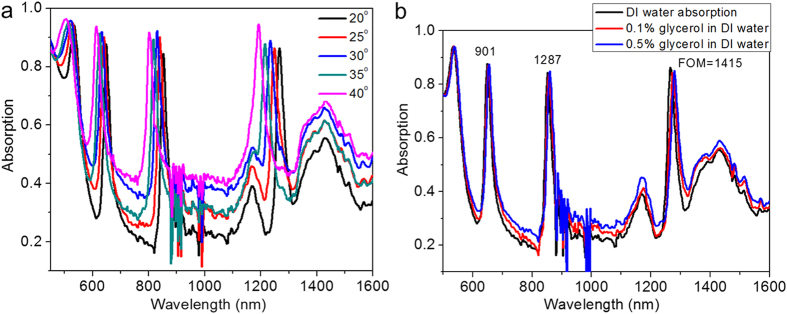
Absorption spectra of Au GC-Au/Al_2_O_3_ HMM and plasmonic absorption sensor. (**a**) Narrow-band near-perfect absorption at different angles of incidence under DI water environment, and (**b**) Refractive index sensing of proposed plasmonic absorption sensor using different weight percentage of glycerol in DI water. The obtained FOM of modes at 1300 nm, 880 nm and 680 nm wavelengths are 1415, 1287, and 901, respectively.

While having a broadband absorber is desirable for many applications there is little effort towards designing ultra-narrow band absorbers. Realizing ultra-narrow band absorption is challenging due to the high optical losses associated with metals in general. In this context, here we propose an ultra-narrow band absorber using Au GC-HMMs, which exhibits for the first time multi-ultra-narrowband absorption in the visible as well as the infrared regimes. Following the same phenomenological reasoning presented earlier, the permittivity contrast between the 20 nm Au grating and the Au-Al_2_O_3_ HMM is much smaller as well as its lower grating modulation amplitude allow only for very narrow modes. Figure 5a shows the absorption spectra of Au GC-HMM for different incident angles (under distilled water (DI) environment), which provides four narrowband absorption peaks based on BPP modes. For 40° angle of incidence, the first mode at 1192 nm has absorption maximum of 95% and 23 nm FWHM, the second mode at 805 nm has absorption maximum of 92% and 18 nm FWHM, and the third mode (614 nm) has absorption maximum of 94% and 17 nm FWHM.

To prove the relevance of Au GC-HMM absorbers, we demonstrate its application as a plasmonic absorber sensor. These sensors rely on the fact that a small change in the environment results in measurable changes in the optical properties of the sensor. In this case, the absorptivity characteristics would change, indicating a change in the polarizability of the superstrate. The figure of merit (FOM) for an absorber plasmonic sensor is given by^37^, 
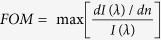
 where 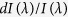 is the relative intensity change at a fixed wavelength induced by a refractive index change *dn*. The maximum FOM reported previously was 24 (refs 36,37 and 87). In order to investigate the sensitivity of our absorber to its environment, we fabricated a microfluidic channel on top of our system (see Methods). We then used distilled (DI) water and mixed it with different concentrations of glycerol which changes the refractive index of water and has tabulated values for the adjusted refractive indices. Figure 5b shows the shift in the absorption peak for 0.1% and 0.5% of glycerol concentrations in DI water. A large wavelength shift of 14 nm, 8 nm and 6 nm are recorded at 20^o^ angle of incidence for the first (1290 nm), second (870 nm) and third (660 nm) modes of 0.5% of glycerol with respect to DI water, respectively and the corresponding refractive index sensitivity are 23,333 nm/RIU, 13,333 nm/RIU and 10,000 nm/RIU. The measured FOM values of our sensor are 1415, 1287, and 901 for the first, second and third mode respectively. It shows that the FOM has a16-fold increase compared to previously reported system^36^.

### Discussion

The absorption spectra of GC-HMMs (Figs 3a and 5a) are significantly changed with incident angle. In particular, all four modes are blue shifted differently with increase in angles of incidence (see Supplementary Fig. S8). This blue shift is attributed to the variation in modal index (effective index) of BPP modes with incident angle. Specifically, the excitation of BPP modes occurs at higher resonance angles when the excitation wavelength in all four BPP wavelength bands decreases (see Supplementary Fig. S9). That means absorption peak wavelength blue shift with increase in angles of incidence. Also note that a smaller resonance angle variation is required to excite the BPP modes of longer wavelength band modes whereas a higher resonance angle variation is necessary for shorter wavelength band modes. This is the reason why blue shift decreased when the BPP mode wavelength is deceased from longer wavelength band mode to shorter wavelength band mode. Furthermore, the change in absorption peak intensity with incident angle is due to the fact that the maximum BPP excitation occurs at different incident angle in each BPP mode band. It should be noted that the maximum change in intensity does not take place at the local maxima of the absorption modes, rather it occurs at a nearby wavelength where the slope of the mode is highest. For the presented sensing scheme, ultra-narrow band absorber represent a remarkable feature for new generation biosensors. In addition, plasmonic absorber sensors offer a valuable advantage; one only needs to use a single wavelength and a single detection angle to perform the sensing measurement, since the detection is based on strong intensity variation for a specific wavelength at a specific angle of incidence. Furthermore, the narrow banded absorption sensor being able to operate at optical frequencies could revolutionize the current biodetection approaches, since binding events could be directly visualized for the remarkable change of the detection signal.

In summary, we designed and fabricated GC-HMM geometries for broadband perfect absorption and ultra-narrowband near-perfect absorption. In particular, these properties have been harnessed to design a sensing system that exhibits polarization independent, wide angle and multiband absorption from visible to near-IR and mid-IR. GC-HMM offers an easy fabrication approach to achieve multi-mode absorption. We demonstrated a plasmonic sensor using narrowband GC-HMM absorber, characterized by a very high FOM (about 1415) which represent a 16-fold increase with respect to the state of the art. The presented GC-HMM absorption based sensor can find a wide range of potential applications such as selective thermal emitters, micro-bolometers, actively integrated photonic circuits, microwave-to-infrared signature control, and for enhancing the performance of photovoltaic and thermo-photovoltaic cells, imaging, absorption filters and plasmonic biosensors.

## Methods

### Device fabrication

HMMs were produced by the sequential deposition of (Al_2_O_3_ and Au layers) and (TiO_2_ and Ag layers) on a glass substrate (Micro slides, Corning) using electron beam evaporation of Al_2_O_3_ and TiO_2_ pellets and thermal evaporation of Au and Ag pellets (both from Kurt J. Lesker Co.). The deposition rates of dielectrics and metals were set to be 0.5 A/s and 0.3 A/s, respectively. 2D metal (Au and Pd) diffraction gratings were realized on top of the HMM by electronbeam lithography (Tescan Vega). Initially, a methyl methacryllate (MMA) resist (8.5MMAEL 11, MICROCHEM) was spin coated on the sample at 4000 rpm and baked at 180 °C for 5 min. A PMMA resist (950PMMA C2 Resist, MICROCHEM) was later spin coated at 5000 rpm and baked at 180 °C for 8 min. The prepared samples were patterned by electron-beam lithography at a dosage of 150 mC/cm^2^ and a beam intensity of 8. The exposed samples were developed using methyl isobutyl ketone (MIBK) and isopropyl alcohol (IPA) solution for 90 s, and IPA for 30 s. Thin films of metal (20 nm thick Au and 8 nm thick Pd) were then deposited directly on top of the sample by the thermal evaporation of Au and Pd pellets as above.

### Microfluidic channel fabrication and integration with the GC-HMM

PMMA caps were prepared by laser micromachining an inlet and outlet (diameter 0.61 mm, separation 12.4 mm) using a VersaLASER system (Universal Laser Systems Inc., Scottsdale, AZ). Double-sided adhesive film (iTapestore, Scotch Plains, NJ) was machined to encompass the PMMA component and 14 × 2 mm microchannels 50 μm in height. The film was attached to the PMMA component to include the inlet and outlet between the outline of the channels. The GC-HMM substrate was then assembled with the PMMA–film structure to form microfluidic channels within the sensing device.

### Optical characterizations

Variable angle spectroscopic ellipsometry (J. A. Woollam Co., Inc, V-VASE) was used to determine the thicknesses and optical constants of the Au, Pd, TiO_2_ and Al_2_O_3_ thin films. The reflection and transmission spectra as a function of excitation wavelengths were acquired using the same instrument with a wavelength spectroscopic resolution of 2 nm.

### Numerical simulations

Finite difference time domain (FDTD) method has been used to simulate the field distributions of GC-HMMs. The commercially available Lumerical FDTD software was used for this purpose. In the FDTD numerical simulations, the Bloch boundary condition with smallest spatial grid size of 1 nm was used for the iteration to maintain the accuracy and stability. Matlab code was used to simulate the effective medium approximation simulations.

## Additional Information

**How to cite this article**: Sreekanth, K. V. *et al.* A multiband perfect absorber based on hyperbolic metamaterials. *Sci. Rep.* **6**, 26272; doi: 10.1038/srep26272 (2016).

## Supplementary Material

Supplementary Information
