# Supplementary material for: A multiband perfect absorber based on hyperbolic metamaterials
Source: Sci Rep. 2016 May 18;6:26272. doi: 10.1038/srep26272 (PMC4870685; doi:10.1038/srep26272)
Supplement: Supplementary Information [file srep26272-s1.pdf]

Supplementary Information: A multiband perfect absorber based on hyperbolic metamaterials

Kandammathe Valiyaveedu Sreekanth[1$*], Mohamed ElKabbash[1$], Yunus Alapan[2], Alireza R. Rashed[1], Umut A. Gurkan[2, 3, 4, 5] and Giuseppe Strangi[1, 6*]

[1]Department of Physics, Case Western Reserve University, 10600 Euclid Avenue, Cleveland, OH, 44106 (USA)

[2]Case Biomanufacturing and Microfabrication Laboratory, Mechanical and Aerospace Engineering Department, Case Western Reserve University, Cleveland, OH, 44106, USA

[3]Biomedical Engineering Department, Case Western Reserve University, Cleveland, OH, 44106, USA

[4]Department of Orthopaedics, Case Western Reserve University, Cleveland, OH, 44106, USA

[5]Advanced Platform Technology Center, Louis Stokes Cleveland Veterans Affairs Medical Center, Cleveland, OH, 44106, USA

[6] Department of Physics and CNR-NANOTEC UOS of Cosenza, Licryl Laboratory, University of Calabria, 87036 - Rende (Italy)

[$] These authors contributed equally

*Correspondence author E-mail: giuseppe.strangi@case.edu, sreekanth3@gmail.com

Fabricated samples

The schematic representation of the fabricated GC-HMM and reference samples are shown in Figure S1: (a) Au diffraction grating on 8 pairs of Au (15 nm)-Al_2O_3 (30 nm) HMM, (b) Pd diffraction grating on 6 pairs of Ag (20 nm)-TiO_2 (20 nm) HMM, (c) Au diffraction grating on a Al_2O_3 (10 nm)/glass substrate and (d) Pd diffraction grating on a TiO_2 (10 nm)/glass substrate. The fabricated microfluidic channel integrated GC-HMM sensor device is shown in Fig. S1e.

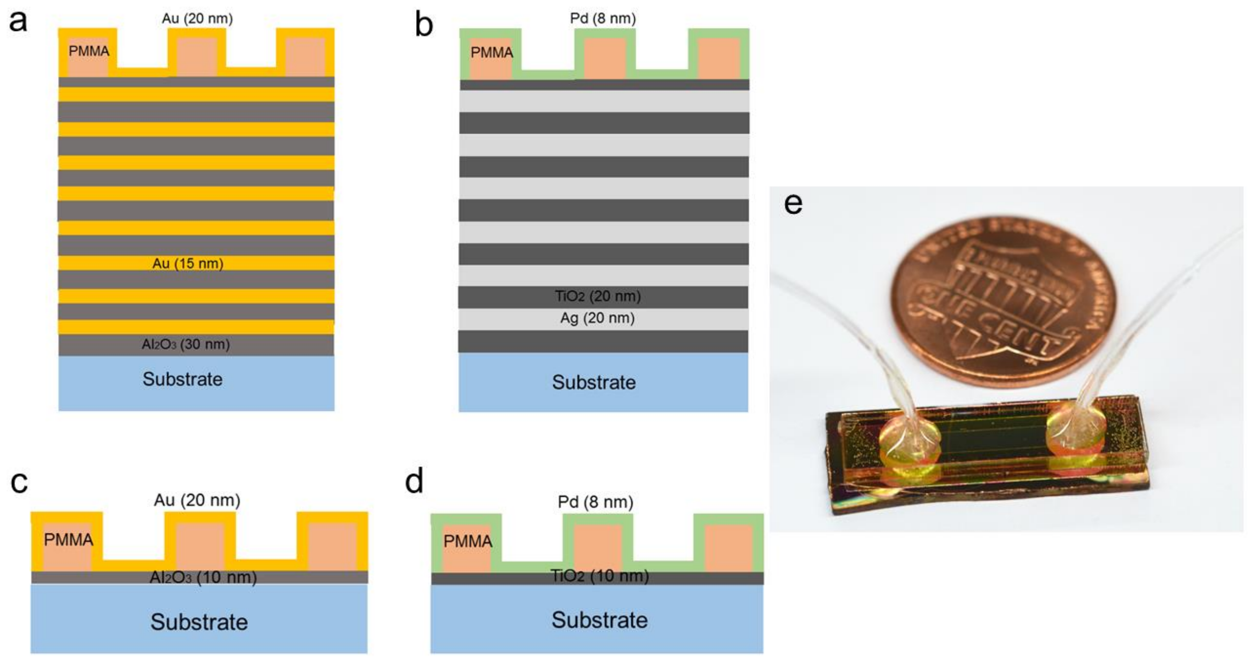

Figure S1 Fabricated samples: (a & b) grating coupled HMMs, (c& d) reference samples and (e) Photograph of fabricated microfluidic channel attached GC-HMM sensor.

Optical constants of Au, Ag, TiO_2 and Al_2O_3

Variable angle spectroscopic ellipsometry (J. A. Woollam Co., Inc, V-VASE) has been used to obtain the optical constants of Au, Ag, TiO_2 and Al_2O_3 thin films. By using thermal evaporation of Au and Ag pellets, Au and Ag thickness of 20 nm was deposited on a glass substrate and a general oscillator model was used to fit the measured ellipsometry data. For the measurements of Al_2O_3 and TiO_2 thin films, 50 nm layer of Al_2O_3 and TiO_2 were first deposited on a silicon wafer

using electron-beam evaporation. A Cauchy model was used to fit the measured data to obtain the refractive index of Al_2O_3 dielectric layer. However, a general oscillator model was used to fit the measured ellipsometry data of TiO_2. The obtained permittivity values of (Au and Al_2O_3) and (Ag, and TiO_2) thin films are shown in Figure S2a and S2b, respectively. Due to Drude-type response of free electrons in metals at higher wavelengths the real permittivity values of Au and Ag show a well-known decreasing trend towards strongly negative values.

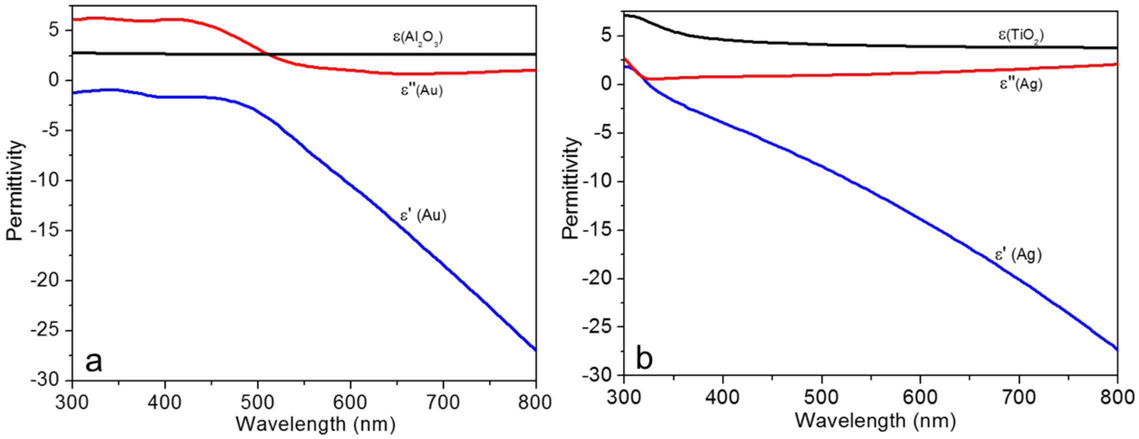

Figure S2 Experimentally determined permittivity values of (a) gold and Al_2O_3, and (b) silver and TiO_2. Blue and red curves represent the real and imaginary values, respectively.

Effective permittivity of HMM

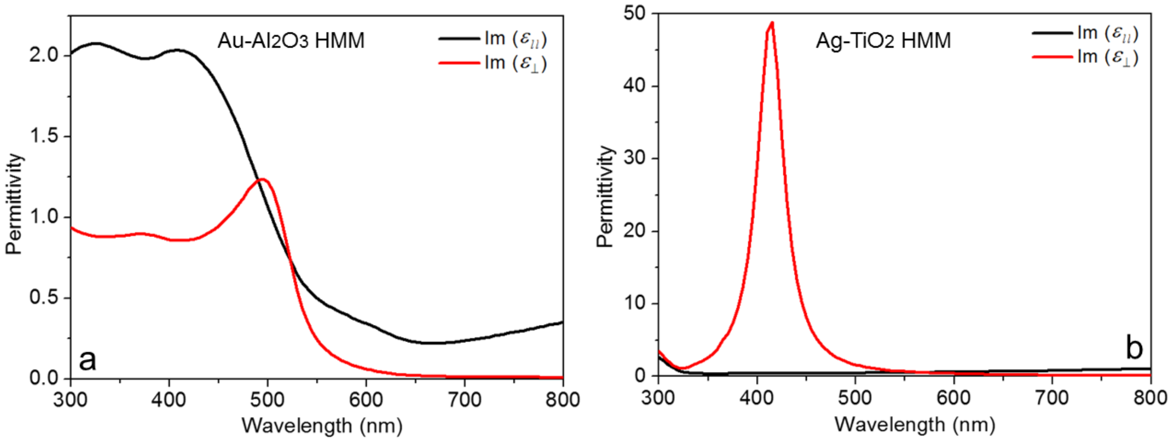

Figure S3 Imaginary effective permittivity values of (a) Au-Al_2O_3 HMM and (b) Ag-TiO_2 HMM

The imaginary effective permittivity values of Au-Al_2O_3 and Ag-TiO_2 HMMs are shown in Figure S3a and S3b, respectively. It is evident from the Figure S3b that the imaginary part of $\varepsilon_\perp$ showing a very sharp Lorentzian shape, peaked exactly at the Type I/Type II transition wavelength (410 nm).

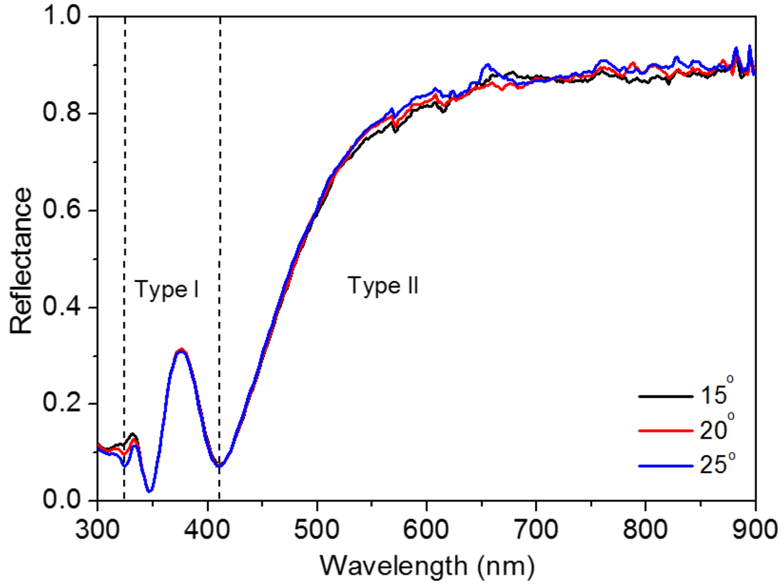

Figure S4 Experimental reflectance spectra of Ag-TiO_2 HMM at different angles of incidence.

For p-polarized light, the reflectance spectra obtained for Ag-TiO_2 HMM at different angles of incidence are shown in Figure S4. One can see that the modes are not shifted when the angle of incidence is varied from 15 to 25 degree. It indicates that those modes are radiative. It is also evident that the reflectance dips obtained at 325 nm and 410 nm wavelengths are exactly matching with transition wavelengths (see Figure 1c), i. e dielectric/Type I at 325 nm and Type I/Type II at 410 nm. Here it is worth noting that the narrow dip present at 325 nm can be attributed to the so called Ferrel-Berreman mode[1]. In our case, the dielectric/Type I transition occurs exactly at the wavelength for which $\varepsilon_\perp = 0$ and the Ag-TiO_2 HMM configures as an epsilon near zero medium. Therefore we can say the occurring of the Ferrel-Berreman mode as a clear signature of the

dielectric/Type I transition. This is the evidence of $\varepsilon_{\perp} = 0$ condition that was previously reported by many groups[1-4].

Effective medium approximations for diffraction gratings

Because the proposed grating structure is entirely different from the deep metallic gratings, it is not possible to model the optical properties of our grating using modeling techniques such as rigorous coupled wave analysis[5], integral equation methods[6] or discrete dipole approximation[7]. Our grating is a two-layer structure (PMMA-metal and metal-air) because it was fabricated by the direct deposition of metal onto a PMMA surface-relief. Therefore we have used effective medium approximations to study the interaction between incident light and the grating. Here, the diffraction grating was approximated as an anisotropic uniaxial medium with effective permittivity in the direction parallel (ε_{ll}) and perpendicular ($\varepsilon_{\perp}$) to the grating features as given by the following equations[8]:

$$\varepsilon_{ll} = f\varepsilon(\omega) + (1-f) \text{ and } \varepsilon_{\perp} = \frac{\varepsilon(\omega)+1+f[\varepsilon(\omega)-1]}{\varepsilon(\omega)+1-f[\varepsilon(\omega)-1]}$$

where $\varepsilon(\omega)$ is the frequency dependent dielectric function of metal and f is the volume fraction occupied by the grating stripes. A similar approach was previously used to simulate the same types of gratings and achieved good agreement with the experimental data [9, 10].

According to this method, the simulated parallel and perpendicular effective real permittivity values of palladium and gold diffraction gratings are shown in Figure S5a and S5b, respectively. The corresponding permittivity values for Ag-TiO_2 and Au-Al_2O_3 HMMs are shown in Figure S5c and S5d, respectively. In the simulations, the complex refractive indices of Ag, Au and Pd and the dielectric constants of TiO_2 and Al_2O_3 are taken from[11]. It is clear from the figures that permittivity (ε_{ll} and $\varepsilon_{\perp}$) contrast between Ag-TiO_2 HMM and 8 nm thick Pd grating is very

high whereas the same between Au-Al_2O_3 HMM and 20 nm thick Au grating is comparatively low. Also, the parallel permittivity contrast increases with increasing wavelength.

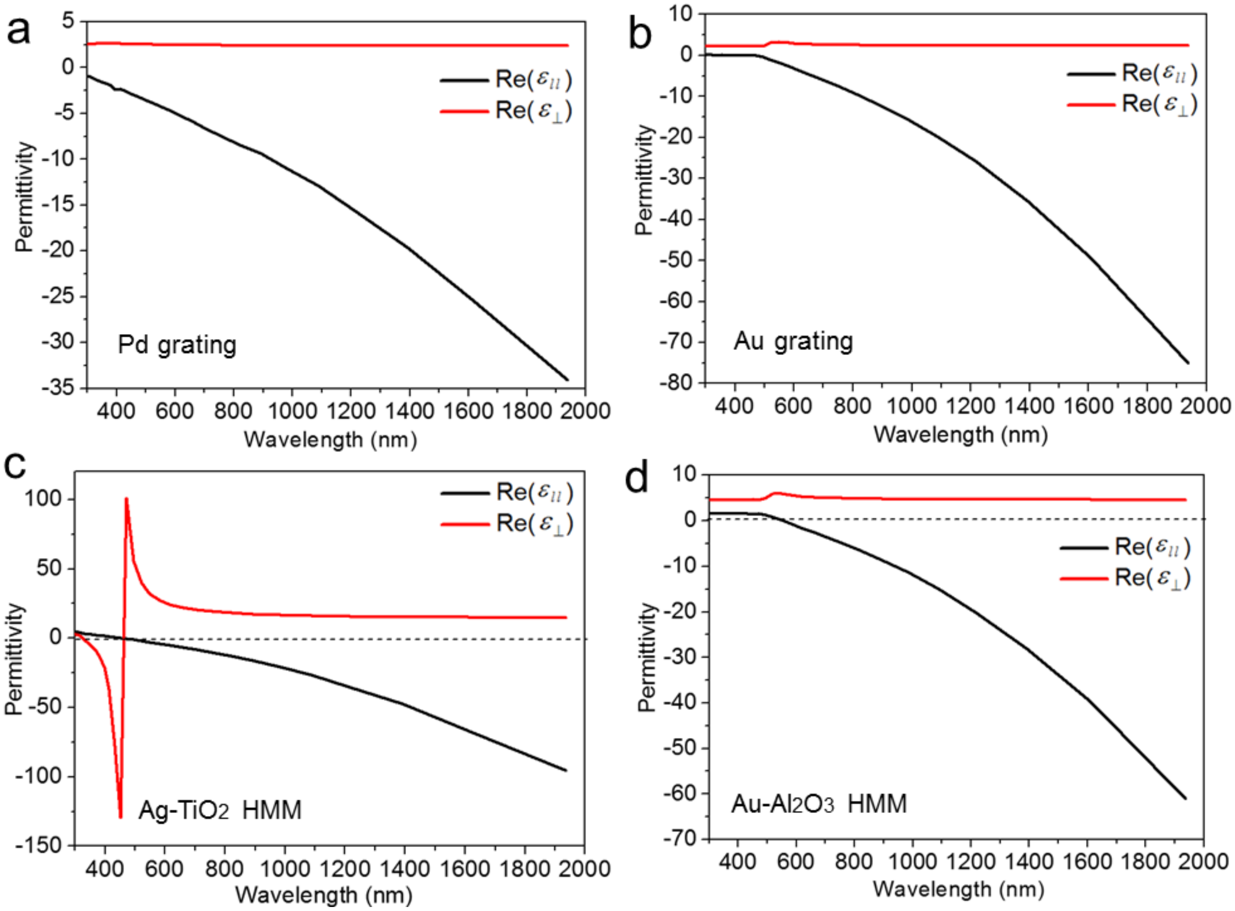

Figure S5 Numerically determined effective permittivity values of (a) Pd grating, (b) Au grating, (c) Ag-TiO_2 HMM and (d) Au-Al_2O_3 HMM.

We observed that the metal used as a grating and its thickness affect the linewidth of the modes. Here we provide a phenomenological explanation for our observations following the guided mode resonance concept[12]. The two main factors affect the modes linewidth: (i) the modulation amplitude of the grating, i.e. $\Delta\varepsilon$ the difference in permittivity between the metal and the dielectric forming the grating. If we took the limit where the deposited metallic layers thickness is much higher than the height of the grating scribed by electron-beam, then the grating modulation

amplitude starts decreasing. This means that the grating starts to vanish as $\Delta\varepsilon$ tends to zero allowing only for narrow band of frequencies to be coupled to the waveguide. Accordingly, having a thinner layer results in a broader mode. (ii) The mode confinement due to high contrast between the grating and the waveguide. As the permittivity of the grating approaches that of the waveguide, the grating remains but the waveguide starts to vanish. Hence it is clear from our results that 8 nm palladium has higher contrast with respect to the effective parallel permittivity of the Ag-TiO_2 HMM compared to the 20 nm gold grating in the Au-Al_2O_3 HMM.

Angular transmission measurements.

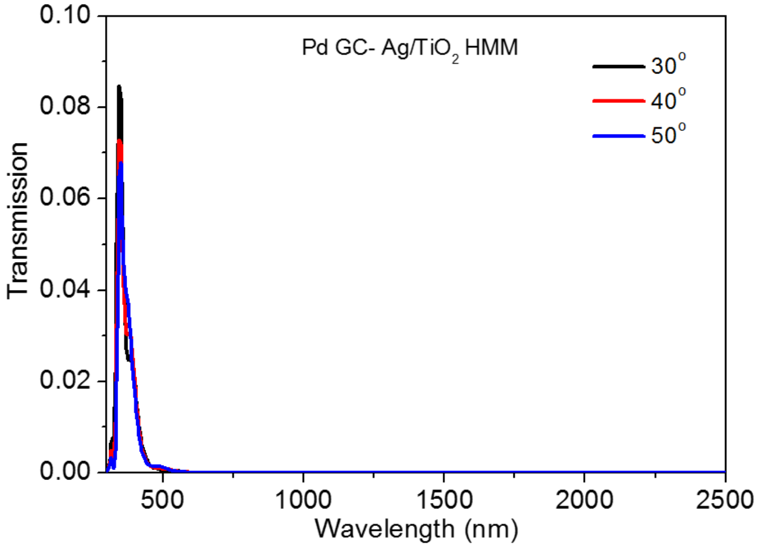

Figure S6 Transmission spectra of Pd GC-HMM at different angles of incidence.

The angular transmission measurements of Pd GC-HMM are shown in Figure S6. It is evident that the transmission intensities are negligible above 420 nm wavelength.

Field distribution maps

Finite difference time domain (FDTD) method has been used to simulate the field distributions of GC-HMMs. The commercially available Lumerical FDTD software was used for this purpose. In the FDTD numerical simulations, the Bloch boundary condition with smallest spatial grid size of 1 nm was used for the iteration to maintain the accuracy and stability. Field distributions were

obtained for both TM and TE polarizations at oblique incidence. As evidenced from the field distributions of Pd GC-HMM (Figure S7), both polarizations show almost same intensity variation inside the HMM for the different mode wavelengths. This further indicates the polarization independent characteristics of our proposed geometries.

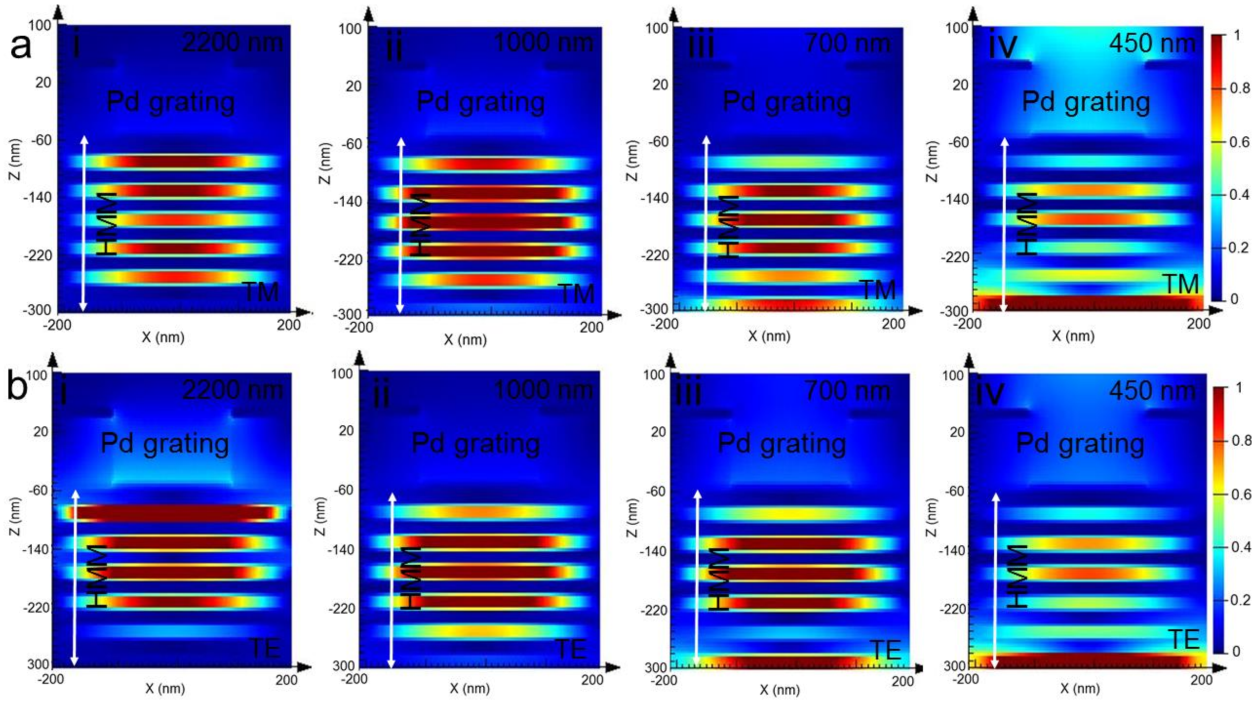

Figure S7 Cross-sectional field distribution maps of Pd GC-HMM at different mode wavelengths: (a) for TM polarization and (b) for TE polarization.

Absorption analysis

The mode blue shift with increasing angle of incidence is due to the variation in modal index (effective index) of BPP modes with incident angle. The absorption change is related to the reflectance of GC-HMM, which in turn is related to the modal index of bulk plasmon polaritons (BPPs). The blue shift of the four modes with incident angle obtained from Figure 3a (for Pd GC-HMM) and Figure 5a (for Au GC-HMM) are shown in Figure S8. In the case of both Pd GC-HMM

and Au GC-HMM, it is evident that the blue shift is higher for longer wavelength band mode and lower for shorter wavelength band mode.

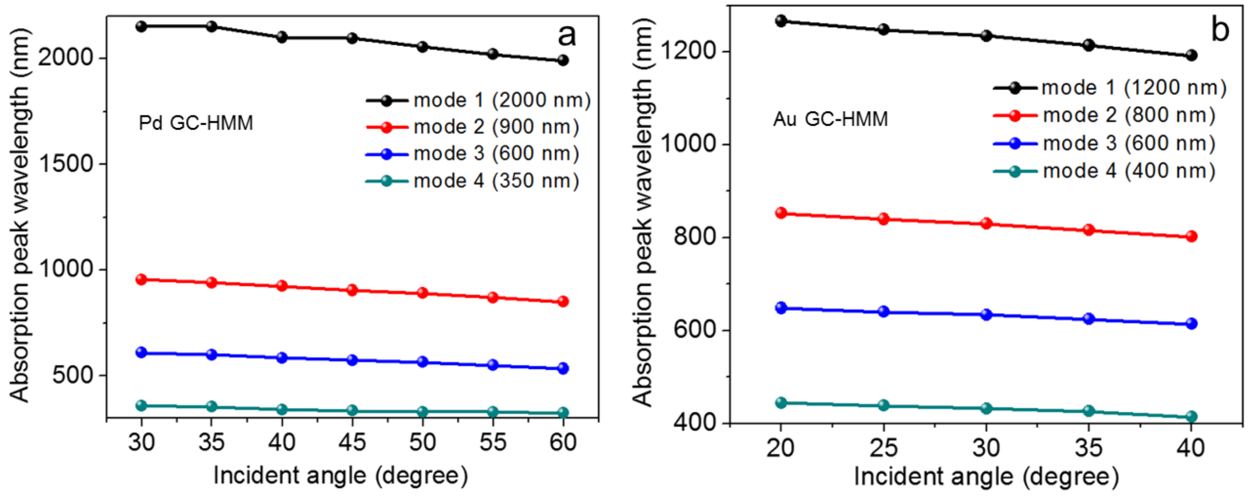

Figure S8 Absorption peak wavelength shift of four BPP modes with angle of incidence: (a) for Pd GC-HMM and (c) for Au GC-HMM.

Since no exact formal theory is available for grating coupling for BPP excitation, we have performed a study on modal index variation with wavelength to experimentally confirm the BPP mode blue shift with incident angle. In order to explain this variation, we study the reflectance spectra as a function of incident angle at a particular wavelength in each BPP mode band. Since Au GC-HMM supports highly confined narrow modes, we consider the reflectance spectrum of Au GC-HMM (Figure 2a). According to the grating coupling technique, the modal index of the mode is given as, $n_{\text{moda}l} = ((\lambda / \Lambda) + n_0 \sin \theta)$, where λ is the incident wavelength, Λ is the grating period, n_0 is the refractive index of incident medium and θ is the incident angle. For a given modal index value, increasing the angle requires decreasing the wavelength, which is translated in a blue shift in our modes. The excitation of bulk plasmon modes at different excitation wavelengths in each BPP band are shown in Figure S9. We investigated the bulk plasmon mode excitation at four

bulk plasmon bands with equal bandwidth such as 2000 to 2050 nm (mode 1), 1000 to 1050 nm (mode 2), 700 to 750 nm (mode 3) and 530 to 580 nm (mode 4) (from Figure 2a). As shown in Figure S9, the resonance angle variation is different in each wavelength band. A decrease in resonance angle is observed when the excitation wavelength in each bulk plasmon band is increased, which shows that the modal index of bulk modes decrease with increase in excitation wavelength. The experimentally calculated BPP modal index values of four bulk plasmon bands are given in Table 1.

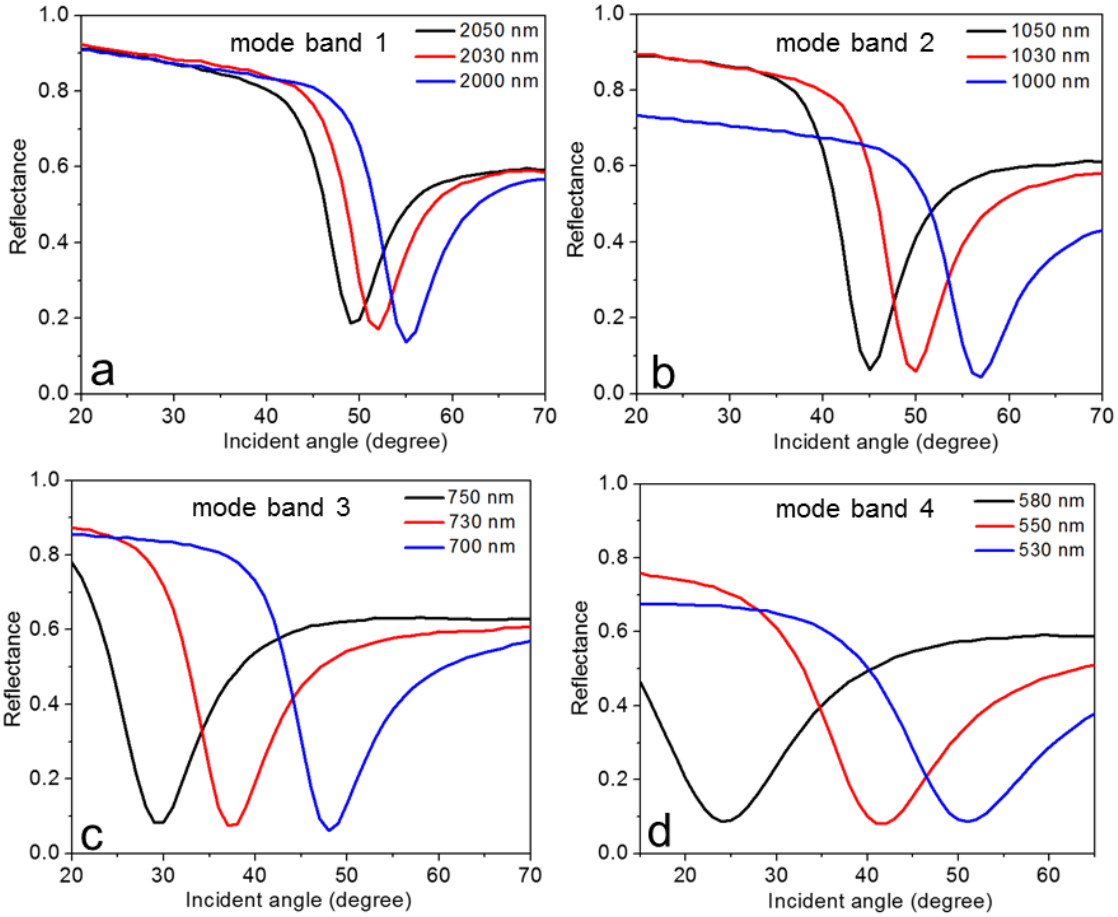

Figure S9 Excitation of bulk plasmon polaritons at different wavelengths in four bulk plasmon bands: for (a) mode band 1, (b) mode band 2, (c) mode band 3 and (d) mode band 4.

The modal indices of BPP modes are given by[13],

$$n_{BPP_N} = \sqrt{\varepsilon_d - \frac{\lambda^2}{\pi^2 t_d t_m}\frac{\varepsilon_d}{\varepsilon_m}} \text{ for N}^{\text{th}} \text{ order mode}$$

$$n_{BPP_0} = \sqrt{\frac{\varepsilon_d \varepsilon_m (t_d + t_m)}{t_d \varepsilon_m + t_m \varepsilon_d}} \text{ for fundamental mode}$$

Wavelength band: 2000 nm to 2050 nm		
Wavelength (nm)	Coupling angle (degree)	Modal index
2000	55	4.82
2030	52	4.84
2050	49	4.85
Wavelength band: 1000 nm to 1050 nm		
Wavelength (nm)	Coupling angle (degree)	Modal index
1000	57	2.84
1030	50	2.83
1050	45	2.80
Wavelength band: 700 nm to 750 nm		
Wavelength (nm)	Coupling angle (degree)	Modal index
700	48	2.19
730	37	2.06
750	29	1.98
Wavelength band: 530 nm to 580 nm		
Wavelength (nm)	Coupling angle (degree)	Modal index
530	51	1.84
550	42	1.77
580	24	1.56

Table 1: Modal index of BPP modes

Where (ε_d , t_d) and (ε_m , t_m) are the dielectric constant and thickness of dielectric and metal, respectively and λ is the wavelength. We then calculated the modal indices of Nth (higher) order and fundamental BPP modes as a function of wavelength. As shown in Figure S10, the modal index of BPP modes decrease with increasing wavelength at shorter wavelengths whereas the modal index of BPP modes slightly vary or constant at longer wavelengths. From the theoretical and experimental data, it is confirmed that the modal index of BPP modes decrease with increasing wavelength for shorter wavelength bands (530 nm to 580 nm, 700 nm to 750 nm and 1000 nm to

1050 nm). However, the modal index values are almost constant at longer wavelength band (2000 nm to 2050 nm).

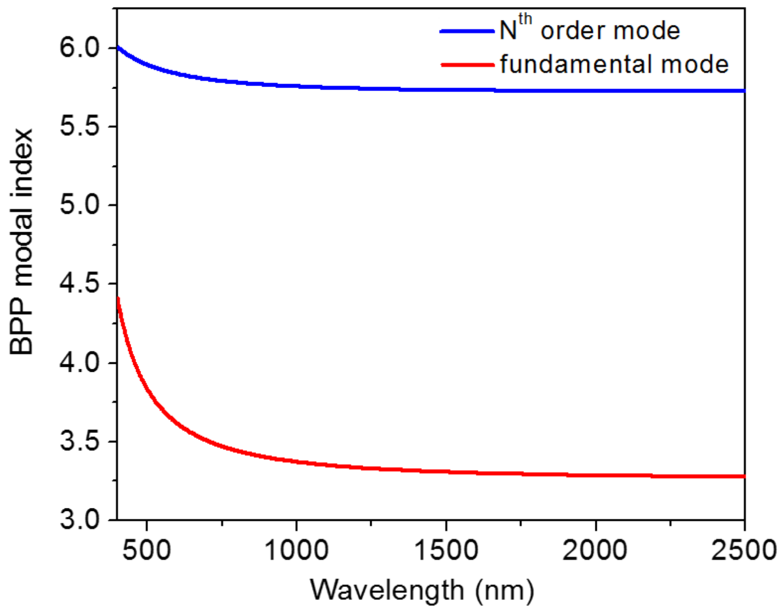

Figure S10 BPP modal index variation with wavelength

On the other hand, the resonance angle increases in each BPP band when the excitation wavelength decreases (Figure S9). That means higher resonance angle is required for the excitation of BPP modes at shorter wavelength of each BPP band. That is why absorption peak (reflection dip) wavelength blue shifted with increase in angle of incidence (Fig. 2 (a, b), Fig. 3a & Fig. 5a in the manuscript). Also note that resonance angle variation increases when the wavelength band of BPP mode decreases. That means a small resonance angle variation of 6 degree is required for the excitation of BPP at mode band 1 (from 2000 nm to 2050 nm). However, mode band 4 (530 nm to 580 nm) required a large resonance angle variation of 27 degree. Therefore, large blue shift is possible for mode band 1 when the incident angle is varied from 35 to 60 deg. This is the reason why blue shift decreased with decrease in BPP mode wavelength band (Figure S8).

References

1. Newman, W. D., et al. Ferrell–Berreman modes in plasmonic epsilon-near-zero media. *ACS Photonics* **2**, 2-7 (2015).

2. Molesky, S., Dewalt, C. J. & Jacob, Z. High temperature epsilon-near-zero and epsilon-near-pole metamaterial emitters for thermophotovoltaics. *Opt. Express*. **21**, 110 (2013).

3. Shekhar, P., Atkinson, J. & Jacob, Z. Hyperbolic metamaterials: fundamentals and applications. *Nano Convergence* **1**, 14 (2014).

4. Hoffman, A. J., et al. Negative refraction in semiconductor metamaterials. *Nat. Mater*. **6**, 946-950 (2007).

5. Moharam, M. G. & Gaylord, T. K. Rigorous coupled-wave analysis of metallic surface-relief gratings. *J. Opt. Soc. Am. A* **3**, 1780 (1986).

6. Mashev, L. B., Popov, E. & Loewen, E. G. Brewster effects for deep metallic gratings. *Appl. Opt.* **28**, 2538 (1989).

7. Gomez-Medina, R. Laroche, M. & Sáenz, J. J. Extraordinary optical reflection from sub-wavelength cylinder arrays. *Opt. Express* **14**, 3730 (2006).

8. Garcia-Vidal, F. J., Pitarke, J. M. & Pendry, J. B. Effective medium theory of the optical properties of aligned carbon nanotubes. *Phys. Rev. Lett.* **78**, 4289 (1997).

9. Kravets, V. G., Schedin, F. & Grigorenko, A. N. Plasmonic blackbody: Almost complete absorption of light in nanostructured metallic coatings. *Phys. Rev. B* **78**, 205405 (2008).

10. Kravets, V. G. Neubeck, S. & Grigorenko, A. N. Plasmonic blackbody: Strong absorption of light by metal nanoparticles embedded in a dielectric matrix. *Phys. Rev. B* **81**, 165401 (2010).

11. Palik, E. D. *Handbook of optical constants of solids*. (Academic Press, 1985**)**.

12. Wang, S. S. & Magnusson, R. Theory and applications of guided-mode resonance filters. *Appl. Opt.* **32**, 2606-2613 (1993).

13. Avrutsky, I., Salakhutdinov, I., Elser, J. & Podolskiy, V. Highly confined optical modes in nanoscale metal-dielectric multilayers. *Phys. Rev. B* **75**, 241402 (2007).
